# *Lawsonella clevelandensis*: a normal flora that bites deep

**DOI:** 10.3389/fcimb.2026.1914466

**Published:** 2026-08-18

**Authors:** Yun Jin, Mengxiao Zhou, Yangmin Gao, Xianfeng Zhou, Xiaohua Tao

**Affiliations:** 1 Jiangxi Provincial Clinical Research Center for Skin Diseases, Dermatology Hospital of Jiangxi Province, Nanchang, China; 2 JXHC Key Laboratory of Skin Infection and Immunity, The Affiliated Dermatology Hospital of Nanchang University, Nanchang, China; 3 Evidence-Based Medicine Research Center, Mass Spectrometry Research Center for Diagnosis, Treatment, and Chronic Disease Rehabilitation, Jiangxi University of Chinese Medicine, Nanchang, China

**Keywords:** 16S rRNA, acid-fast, anaerobic bacteria, emerging pathogen, *Lawsonella clevelandensis*, metagenomics, soft tissue infection

## Abstract

Since its formal description in 2016, *Lawsonella clevelandensis*—a strictly anaerobic, partially acid-fast bacterium—has been increasingly recognized as a cause of deep-seated abscesses, yet its fastidious nature and absence from routine diagnostic databases contribute to significant underdiagnosis. This narrative review synthesizes current knowledge on its microbiology, expanding clinical spectrum, diagnostic strategies, and treatment, based on a literature search of PubMed and Web of Science up to April 2026. Analysis of 27 documented publications, comprising 18 clinical cases, reveals a potential association with host risk factors including diabetes, immunosuppression, and prior surgical procedures, alongside a notable predilection for fat-rich tissues such as the breast and abdomen. While metagenomic next-generation sequencing and 16S rRNA gene amplification have become indispensable for definitive identification, antimicrobial susceptibility data—derived primarily from a single strain—demonstrate uniformly low minimum inhibitory concentrations for penicillins, carbapenems, clindamycin, and metronidazole, with no acquired resistance genes identified by whole-genome sequencing. However, the absence of established clinical breakpoints and limited tested isolates precludes definitive conclusions about universal susceptibility. Clinicians should maintain a high index of suspicion for *L. clevelandensis* in culture-negative deep abscesses, particularly those with acid-fast rods, as prompt diagnosis and empirical therapy with β-lactam/β-lactamase inhibitors or carbapenems appear reasonable based on current *in vitro* and clinical evidence, though further susceptibility surveillance is essential.

## 1 Introduction

Anaerobic bacteria are among the most common causative agents of deep-seated and soft tissue abscesses. Among these, the spectrum of clinically relevant anaerobes has continued to expand with advances in cultivation techniques and, more recently, the widespread application of metagenomic next-generation sequencing (mNGS). *Lawsonella clevelandensis*, formally described as a novel genus and species in 2016 (Bell et al., 2016), exemplifies this phenomenon. Although initially recognized only through molecular methods in a small number of abscesses from multiple medical centers (Harrington et al., 2013), it has since emerged as an increasingly reported cause of diverse human infections, affecting soft tissue, visceral, and even neural sites (Ramesh et al., 2021; Kumaria et al., 2023; Zhou et al., 2023; Lijia et al., 2024; Lindstrom et al., 2025; Hennen et al., 2026).

*L. clevelandensis* is a Gram-variable, strictly anaerobic, partially acid-fast bacterium that is difficult to culture using conventional methods and is absent from most commercial identification databases. These characteristics create substantial diagnostic challenges, and it is widely believed that the organism is significantly underdiagnosed. The advent of mNGS and broad-range 16S rRNA gene amplification has dramatically improved detection rates, leading to a rapid expansion of the documented clinical spectrum over the past five years (Wang et al., 2023; Zhao et al., 2025; Yang et al., 2026). Notably, a brain abscess case was recently reported in the setting of invasive cutaneous squamous cell carcinoma identified via bedside stereotactic aspiration (Hennen et al., 2026), underscoring that the full clinical repertoire of this organism has yet to be fully defined.

**Figure 1:**
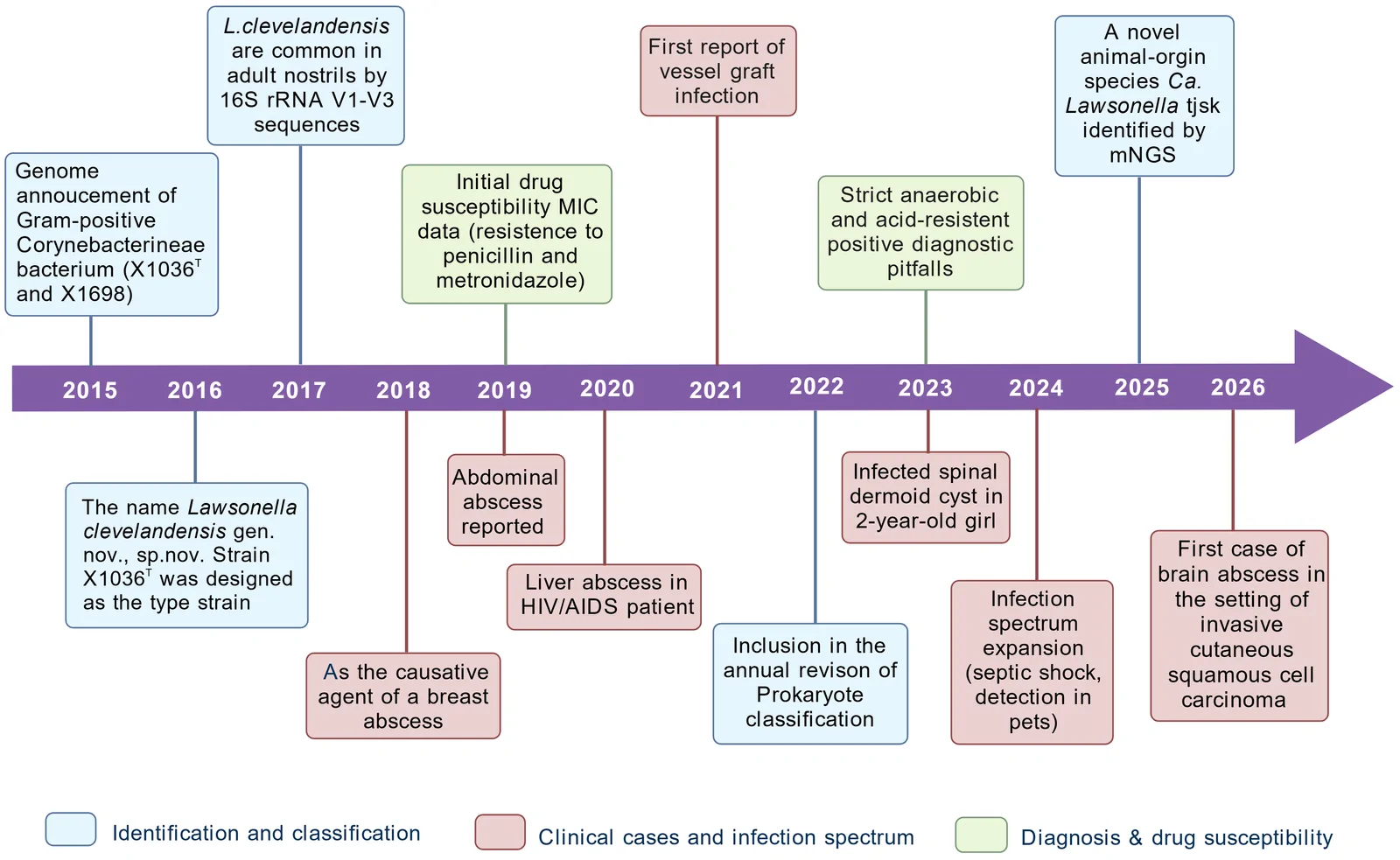
Time line of major events of the identification, clinical spectrum and diagnostic advances of *L. clevelandensis* infections.

This review synthesizes the current state of knowledge on *L. clevelandensis*, covering taxonomy and microbiological characteristics, the expanding clinical spectrum, diagnostic strategies, antimicrobial susceptibility, treatment outcomes, and key directions for future research. The literature search query was formulated as follows: (“Lawsonella clevelandensis” OR “L. clevelandensis” OR “clevelandensis”), and a total of 27 documents were retrieved, including 18 clinial cases (described in 14 documents) described around the globe. A time line of major events in the identification, clinical spectrum and diagnostic advances of *L. clevelandensis*-associated infections is presented in **Figure 1**. This review is intended to serve both clinical microbiologists and infectious disease specialists who may encounter this elusive pathogen in practice.

## 2 Taxonomy and microbiological characteristics

### 2.1 Taxonomic position and discovery

*L. clevelandensis* was formally described as a new genus and species by Bell et al. in 2016, published in the International Journal of Systematic and Evolutionary Microbiology (Bell et al., 2016). The organism was first isolated from human clinical specimens, predominantly from abscess aspirates (Harrington et al., 2013). In the clinical specimen, bacilli were branching, Gram positive, and acid fast. The organism grew slowly and was not identified by 16S rRNA sequencing. Their findings supported the description of a new genus and species of the suborder Corynebacterineae (Harrington et al., 2013).The genus name *Lawsonella* honors Dr. Lawson, and the species epithet *clevelandensis* reflects the Cleveland Clinic in Ohio, United States, where early investigations were conducted. The taxonomic hierarchy is as follows: Order *Corynebacteriales*, Family *Lawsonellaceae* (novel family), Genus *Lawsonella*, Species *L. clevelandensis*. It belongs to the suborder *Corynebacterineae*, sharing a broader phylogenetic neighborhood with the genera *Corynebacterium*, *Dietzia*, *Nocardia*, and *Rhodococcus*. The 16S rRNA gene reference sequence is deposited in GenBank under accession number JX877776.

The complete genome sequences of two strains were obtained using mNGS, and the sequences of X1036^T^ and X1698 have been deposited at GenBank under the accession numbers CP009312 and CP012390, respectively (Nicholson et al., 2015). Genome-based analyses, including average nucleotide identity (ANI) comparisons with closely related taxa, confirmed the distinctiveness of Lawsonella as a separate genus. The genome size is approximately 2.0-2.5 Mb with a G+C content of approximately 58–60 mol%. Notably, a 2025 metagenomics study identified a novel candidate species within the genus, designated ‘*Candidatus Lawsonella tjsk*,’ represented by 43 metagenome-assembled genomes (MAGs) from human sources (Zhao et al., 2025), suggesting that the genus *Lawsonella* may harbor additional undescribed diversity with clinical relevance.

### 2.2 Microbiological phenotype

*L. clevelandensis* is a Gram-variable, strictly anaerobic, partially acid-fast bacterium that is difficult to culture using conventional methods and is absent from most commercial identification databases. Its microbiological characteristics is summarized in **Table 1**.

**Table 1:** The microbiological characteristics of *L. clevelandensis*.

Feature	Description
Gram stain	Variable (Gram-positive to Gram-negative depending on culture conditions)
Acid-fast stain	Partially acid-fast (key diagnostic clue; may mimic Mycobacterium)
Oxygen requirement	Strictly anaerobic
Growth rate	Extremely slow; requires ≥5–14 days of anaerobic incubation
Colony morphology	Small, white, non-hemolytic
Catalase	Positive
Oxidase	Negative
Chemotaxonomy	Contains mycolic acids; meso-diaminopimelic acid (meso-DAP) in peptidoglycan
Metabolism	Obligate anaerobe; fermentative metabolism

The partial acid-fast property is a particularly noteworthy feature, as it may lead clinicians and microbiologists to initially suspect *Mycobacterium tuberculosis* or nontuberculous mycobacteria (NTM), resulting in inappropriate antitubercular therapy before the correct diagnosis is established. The slow growth rate and strict anaerobic requirements further contribute to the organism’s tendency to be missed or misidentified in routine clinical laboratories.

## 3 Clinical spectrum and epidemiology

### 3.1 Documented infection types

**Figure 2:**
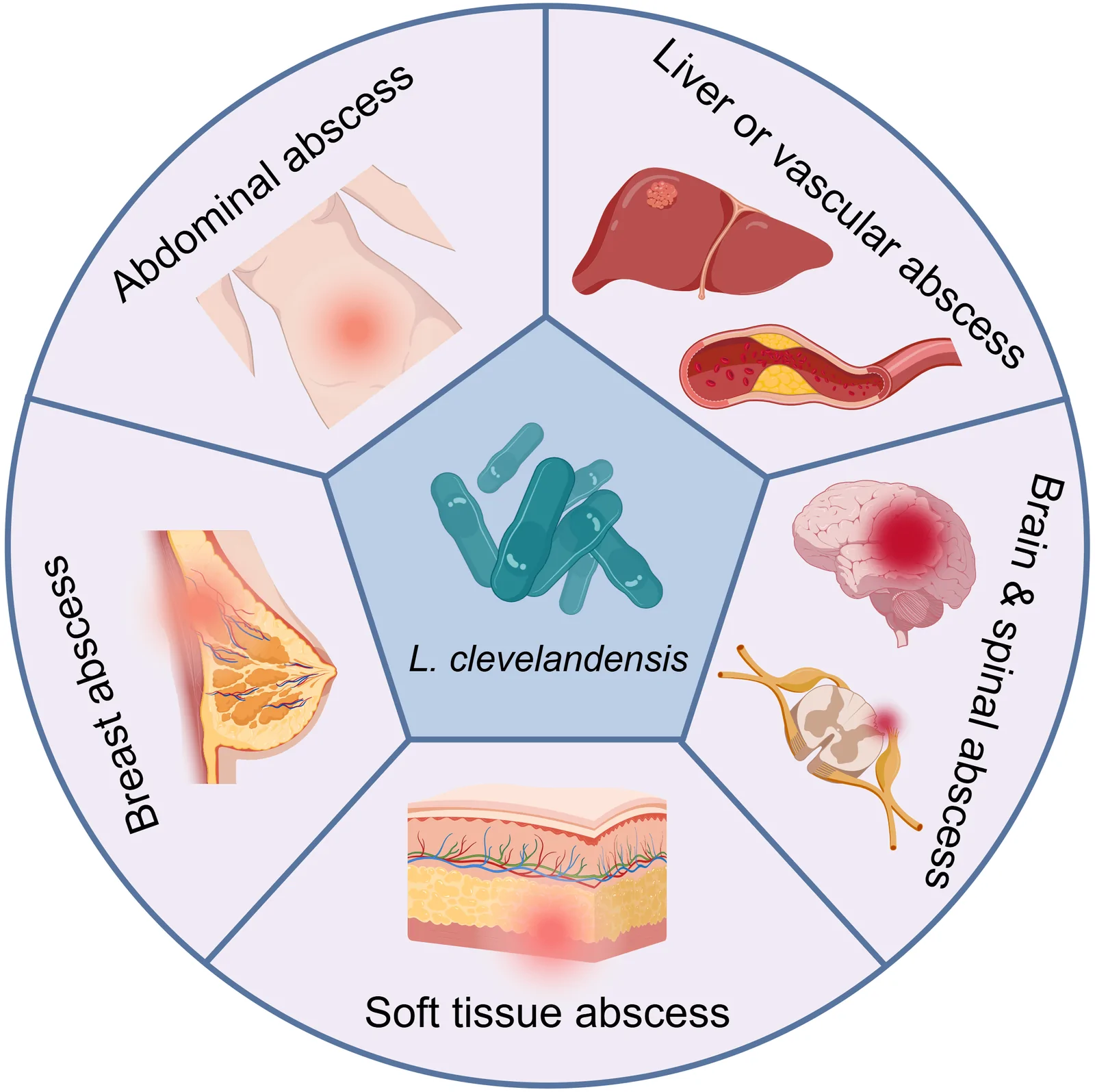
Organ or tissue affected by abscesses associated with *L. clevelandensis*.

Since its initial description, *L. clevelandensis* has been implicated in an expanding range of human infections, affecting a wide range of organs or tissues (**Figure 2**). A comprehensive review of the literature reveals infections across multiple anatomical sites, with a striking predilection for fat-rich soft tissues. The clinical entities reported to date are summarized in **Table 2**.

**Table 2:** The clinical cases (n=18) reported globally from 2013 to 2026.

Infection type	Host background	Age/sex	Location	Clinical features	Sample	Diagnosis	Antibiotic therapies	Prognosis	Ref
Spinal abscess	Metastatic prostate cancer	65y/M	USA	An enlarging mass adjacent to a surgical incision at T3–8 from previous spinal stabilization	Tissue, fluid	16S rRNA sequencing	VAN→SAM→MPM→ SXT→AMC	Death associated with prostate cancer	(Harrington et al., 2013)
Breast abscess	A dermoid cyst of the ovary, steatosis of the liver, diabetes mellitus	44y/F	Canada	A left breast abscess in 2003 that was surgically drained and managed with a short course of antibiotic therapy.	Abscess cavity	16S rRNA sequencing	VAN→CLOXA→AMC	Cure	(Harrington et al., 2013)
Breast abscess	Poorly controlled type II diabetes, diabetic foot ulcers, and furunculosis	23y/F	Canada	A 3-month history of a progressively enlarging erythematous left breast lump	Pus	16S rRNA sequencing	CIP+MTZ→SXT	Cure	(Harrington et al., 2013)
Liver abscess	Coronary artery bypass graft; an automatic implantable cardiac defibrillator; aortic stenosis	81y/M	USA	A hypodense mass in the left lobe of the liver with poor enhancement	Aspirate material	16S rRNA sequencing	LVX+MTZ+VAN+CLI+SXT→CLI+SXT →SXT	Cure	(Harrington et al., 2013)
Breast abscess	A family history of breast carcinoma	29y/F	Portugal	A 3-week evolution of a breast nodule	Histological sample	16S rRNA sequencing	SXT→AMC	Cure	(Favila Menezes et al., 2018)
Spinal abscess	Immunocompetent	2y/F	UK	An infected spinal dermoid cyst	Pus	16S rRNA sequencing	CXM+MTZ→AMC→ CXM→LZD	Cure	(Kumaria et al., 2023)
Abdominal abscess	Inflammatory Bowel Disease	38y/M	USA	A history of medically-refractory ulcerative colitis	Percutaneous drain	16S rRNA sequencing	Broad spectrum antibiotics→ anti-TB treatment→AMC	Cure	(Chudy-Onwugaje et al., 2019)
Liver abscess	Rheumatoid arthritis	70y/M	USA	Liver abscesses and pylephlebitis	Liver drainage and ascitic fluid	16S rRNA sequencing	TZP→VAN→MTZ→IPM+MXF+CLR→AMC	Cure	(Gonzales Zamora et al., 2020)
Intra-abdominal abscess	abdominal aortic aneurysm (AAA)	70y/M	UK	CCR-AAA with infected haematoma	Needle aspirate	16S rRNA sequencing	TEIC+MTZ →AMC	Death associated with declining renal function	(Ahmed et al., 2021)
Psoas abscess	Underwent surgical resection and replacement of infected graft	65y/M	USA	Bilateral psoas abscesses secondary to aorto-bi-iliac vascular graft infection	Drainage	16S rRNA sequencing	DTC+ CFR	Cure	(Ramesh et al., 2021)
Vascular graft infection (VGI)	Medical history of AAA status post endovascular stenting repair	65y/F	USA	An enhancing lesion involving the L3–L4 inferior endplates consistent with perivascular abscess and spinal osteomyelitis	Aortic tissue	16S rRNA sequencing	CFPM+MTZ+DAP→DTC→AMC	lifelong oral AMC suppression	(Nour et al., 2023)
Cardiac infections	History of mechanical aortic valve (AV) replacement and aortic annulus patch augmentation	65y/F	USA	Dyspnea, fatigue, and concern for infective endocarditis (IE)	Aortic valve tissue	16S rRNA sequencing	CRO+VAN	Death	(Nour et al., 2023)
Urinary tract infections	Kidney transplant and progressive worsening of kidney function	29y/M	Chile	In the context of a pauci-immune vasculitis which required greater immunosuppression	Urine sample	16S rRNA sequencing	AMC	Cure	(Zavadzki et al., 2023)
Breast abscess	Post-cosmetic procedure of breast augmentation	29y/F	China	Deep soft tissue infection following autologous fat transfer	Subcutaneous necrotic tissue and pus	NGS	TZP+LVX→Cefoxitin+Ornidazole	Cure	(Zhou et al., 2023)
Lung and scrota abscesses	Hidradenitis suppurativa (HS) inducing septic shock	33y/M	China	Underwent drainage of the chest and right hemothorax removal under thoracoscopic	Left neck and scrotal abscess	NGS	TZP+VAN→TZP+MIN→MIN	Cure	(Lijia et al., 2024)
Breast abscess	Diabetes mellitus, end-stage renal disease, and chronic encephalopathy	56y/F	USA	Right breast pain for a week	Viscous purulent fluid	16S rRNA sequencing	CLI →AMC	Cure	(Lindstrom et al., 2025)
Breast abscess	Immuno- competent	46y/F	China	A history of left breast abscess for 6 years	Pus	NGS	TZP→AMC	Cure	(Liu et al., 2025)
Brain abscess	Recurrent, locally invasive facial squamous cell carcinoma	74y/F	USA	Shaking of her lower extremity and a large right frontal mass extending through the calvarium and orbit	Biopsy	NGS/qPCR	LZD+MPM+SXT→ CRO+MTZ	Cure	(Hennen et al., 2026)

Ref, reference; y, years; M, male; F, female; s/p, status post; TB, tubercular; VAN, vancomycin; SAM, ampicillin/sulbactam; MPM, meropenem; SXT, trimethoprim-sulfamethoxazole; AMC, amoxicillin/clavulanic acid; CLOXA, cloxacillin; CIP, ciprofloxacin; MTZ, metronidazole; LVX, levofloxacin; CLI, clindamycin; CXM, cefuroxime; LZD, linezolid; TZP, piperacillin/tazobactam; IPM, imipenem; MXF, moxifloxacin; CLR, clarithromycin; DTC, doxycycline; CFR, cefadroxil; TEIC, teicoplanin; CFPM, cefepime; DAP, daptomycin; CRO, ceftriaxone; MIN, minocycline.

### 3.2 Epidemiology and risk factors

To date, 18 human cases have been documented in the peer-reviewed literature as detailed in **Table 2**, though the true incidence is almost certainly higher due to systematic underdiagnosis. Cases have been reported across North America, Europe, and Asia, indicating a global distribution with no apparent geographic clustering.

Host risk factors include immunosuppression, diabetes mellitus, and conditions associated with impaired skin barrier integrity. Notably, a substantial proportion of cases have occurred in immunocompetent individuals (Favila Menezes et al., 2018; Chudy-Onwugaje et al., 2019; Nour et al., 2023; Zhou et al., 2023), indicating that *L. clevelandensis* can act as an opportunistic pathogen even in the absence of overt immune dysfunction. The organism has been detected as part of the normal skin commensal microbiota (Escapa et al., 2018; Francuzik et al., 2018; Polak-Witka et al., 2021; Wang et al., 2023), suggesting a cutaneous reservoir from which it can invade deeper tissues following trauma, surgery (Kumaria et al., 2023; Zhou et al., 2023; Liu et al., 2025), or in the context of chronic inflammatory skin diseases (Harrington et al., 2013; Bell et al., 2016).

The strong association with fat-rich tissues (breast, abdomen, subcutaneous tissue) may reflect specific microbial virulence traits or ecological advantages conferred by the lipid-rich microenvironment. This tissue tropism parallels observations in other members of the *Corynebacteriales*, several of which are known to metabolize host lipids as a carbon source (Rahlwes et al., 2019). In this small case series, 6 out of 8 adult female cases (75%) developed breast abscess, indicating a potential tissue tropism. These findings also provide evidence and lessons for clinical practice regarding breast abscess management in the future.

### 3.3 Transmission and source

**Figure 3:**
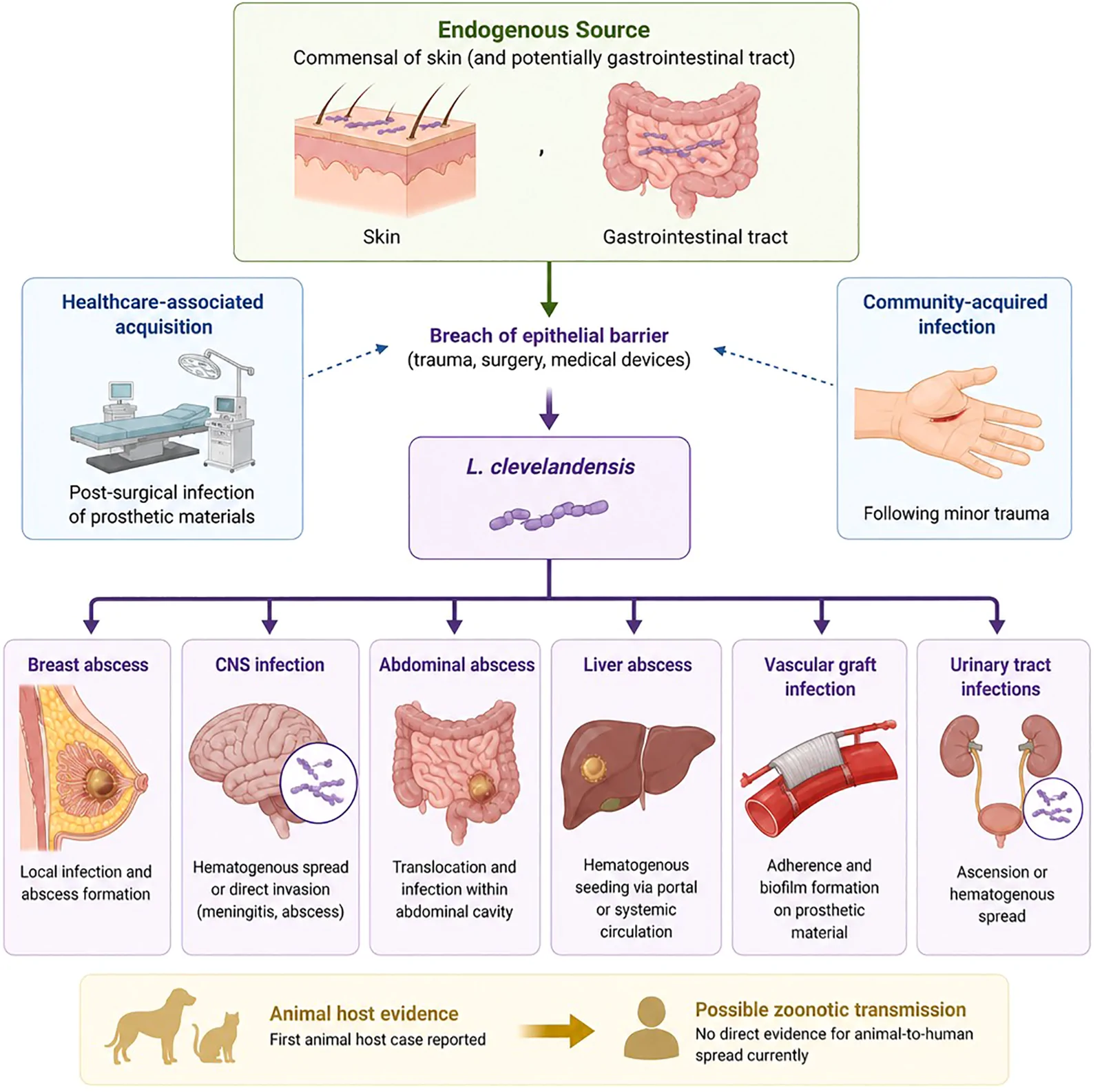
Proposed model for transmission and infection of *L. clevelandensis* from its endogenous skin commensal niche.

The precise transmission route remains incompletely defined. Current evidence supports an endogenous source model in which *L. clevelandensis*, as a commensal of skin (and potentially the gastrointestinal tract) (Francuzik et al., 2018; Zhao et al., 2025), gains access to deeper tissues through breach of the epithelial barrier (Zhou et al., 2023; Lijia et al., 2024) (**Figure 3**). Healthcare-associated acquisition (including post-surgical infection of prosthetic materials) and community-acquired infection following minor trauma have both been documented. The first animal host case raises the possibility of zoonotic transmission (Toth et al., 2025; Zhao et al., 2025), although direct evidence for animal-to-human spread is currently lacking.

## 4 Diagnostic challenges and advances

### 4.1 Limitations of conventional methods

*L. clevelandensis* presents multiple layers of diagnostic challenge. First, its strict anaerobic requirement means it will not grow under standard aerobic or CO_2_-enriched incubation conditions (Harrington et al., 2013; Nicholson et al., 2015). Even when appropriately cultured anaerobically, its slow growth necessitates incubation periods of at least 5–14 days — far longer than the 3–5 day incubation typical for most clinical anaerobic cultures. Second, its variable Gram stain appearance does not provide a consistent clue, and the partial acid-fast property frequently misleads laboratories into pursuing a mycobacterial diagnosis, resulting in treatment delays (Chudy-Onwugaje et al., 2019). Third, and critically, *L. clevelandensis* is not included in the reference spectra of commercially available matrix-assisted laser desorption/ionization time-of-flight mass spectrometry (MALDI-TOF MS) systems (including Bruker Biotyper and VITEK MS). As a result, even when the organism is successfully isolated, routine MALDI-TOF identification fails, often returning as “no reliable identification.” Whole-genome sequencing (WGS) (Nicholson et al., 2015; Goldenberger et al., 2019) and 16S rRNA gene sequencing have been used to resolve the identification in such cases (Gonzales Zamora et al., 2020; Ahmed et al., 2021; Ramesh et al., 2021; Lijia et al., 2024).

To facilitate accurate differentiation, we provide key differentiating points for laboratory and clinical practice:

- Oxygen requirement is the most rapid and practical differentiator: *L. clevelandensis* grows only under anaerobic conditions, whereas all clinically significant mycobacteria are aerobic. Failure to grow on aerobic mycobacterial media together with growth on anaerobic media strongly suggests *L. clevelandensis.*
- Acid-fast intensity: *L. clevelandensis* is typically partially or weakly acid-fast, whereas *M. tuberculosis* and mostnontuberculous mycobacterial (NTM) are strongly acid-fast. If acid-fast bacilli are observed but staining is variable or weak, *L. clevelandensis* should be included in the differential.
- Antimicrobial response: *L. clevelandensis* responds to β lactam/β lactamase inhibitors, carbapenems, and clindamycin—agents generally inactive against mycobacteria. Lack of response to anti tuberculous therapy should prompt reconsideration of the diagnosis.

### 4.2 Molecular diagnostic approaches

In addition to prolonged incubation, culture-based identification of anaerobic bacteria is inherently unreliable. Phenotypic methods often yield ambiguous or false identifications, with many anaerobes being nonreactive in commercial diagnostic systems. Even when growth is achieved, conventional identification systems may misidentify or fail to identify unusual or emerging pathogens. Molecular methods that do not require prior isolation have become the cornerstone of *L. clevelandensis* diagnosis. They circumvent prolonged culture and overcome the inherent limitations of phenotypic identification.

#### 4.2.1 16S rRNA gene amplification and sequencing

Broad-range PCR amplification of the 16S rRNA gene followed by Sanger sequencing remains the most widely available molecular confirmatory method (Gonzales Zamora et al., 2020; Ahmed et al., 2021; Ramesh et al., 2021; Lijia et al., 2024). A 704-1,500 bp amplicon can be sequenced and compared against reference databases (e.g., GenBank). A nearly 100% sequence identity match to the *L. clevelandensis* reference (JX877776) is considered confirmatory. This approach is particularly valuable when MALDI-TOF fails or when only stained smears are available from paucibacillary specimens.

#### 4.2.2 Metagenomic next-generation sequencing

Clinical mNGS has emerged as the most powerful tool for detecting *L. clevelandensis*, especially in culture-negative infections (Navas et al., 2016; Wang et al., 2023; Lijia et al., 2024; Zhang et al., 2025; Yang et al., 2026). Multiple recent case reports — including the 2024 lung and scrotal abscess case, the 2025 post-liposuction case, and the 2026 brain abscess case — were all first diagnosed by mNGS. mNGS provides species-level identification directly from clinical specimens (tissue, pus, abscess fluid, cerebrospinal fluid) without the need for culture, making it particularly suited to fastidious or slow-growing organisms.

#### 4.2.3 Whole-genome sequencing

WGS provides the highest-resolution identification and has also been used to identify potential virulence genes and predict antimicrobial resistance profiles (Goldenberger et al., 2019). With decreasing costs and increasing accessibility, WGS is likely to play a growing role in both clinical diagnosis and epidemiological surveillance of *L. clevelandensis* infections.

### 4.3 Recommended diagnostic algorithm

**Figure 4:**
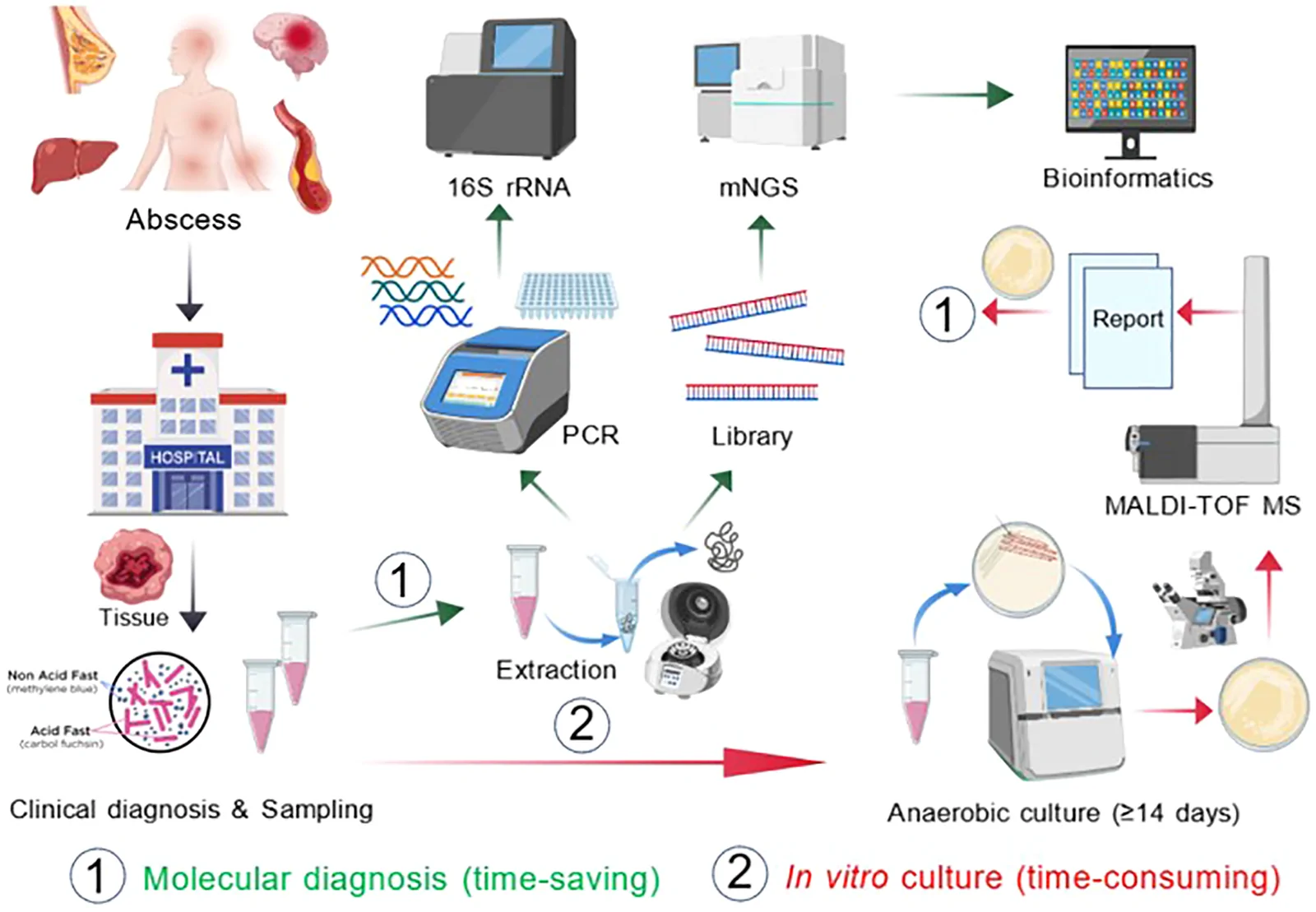
Diagnostic approaches for suspected infections associated with *L. clevelandensis.* Given the prolonged culture time (5–14 days) and low recovery rate of this fastidious anaerobe, molecular methods—particularly mNGS and 16S rRNA gene sequencing—are recommended as first-line diagnostic tools for timely pathogen identification. Concurrently, conventional bacterial culture should still be performed in parallel, including both aerobic and anaerobic incubation, and maintained for at least 14 days before being discarded as negative, to maximize the chance of isolate recovery for subsequent susceptibility testing.

Based on the current literature, the following stepwise diagnostic approach is proposed for suspected *L. clevelandensis* infection (**Figure 4**):

Step 1: Consider the diagnosis in patients presenting with culture-negative deep soft tissue or visceral abscesses, particularly in fat-rich locations (breast, abdomen, subcutaneous tissue).
Step 2: Perform acid-fast staining. If positive, consider both mycobacteria and *L. clevelandensis* in the differential diagnosis — do not initiate antitubercular therapy without excluding this organism.
Step 3: Proceed directly to 16S rRNA PCR and sequencing from the primary specimen. mNGS is also recommended for molecular identification of mono-infection or co-infection.
Step 4: Initiate extended anaerobic culture (≥14 days) in parallel with standard cultures. If MALDI-TOF MS fails to identify the organism, or for culture-negative cases, critically ill patients despite empiric therapy, apply clinical mNGS on the primary specimen. mNGS is particularly recommended for brain abscess, vascular graft infection, and post-surgical deep infections.
Step 5: Report positive findings to clinical teams promptly and initiate targeted antimicrobial therapy (see Section 5).

## 5 Treatment and antimicrobial susceptibility

### 5.1 Clinical practice in treatment

Given the acid-fast and Gram-positive staining properties of *L. clevelandensis*, empirical antibiotic regimens in reported cases have often targeted both anaerobes and mycobacteria. As summarized in **Table 2**, the most commonly used agents include meropenem, amoxicillin-clavulanate (AMC), and trimethoprim-sulfamethoxazole (SXT), among others. Notably, AMC—which has good activity against most anaerobes—was successfully used in the majority of patients, either as initial therapy or after step-down from intravenous regimens. For example, patients with spinal abscess and breast abscess showed substantial clinical improvement following AMC treatment. However, the choice of antibiotics in these cases was largely empirical, driven by the organism’s staining characteristics and the clinical presentation of deep abscesses. It is important to recognize that the only comprehensive antimicrobial susceptibility data available to date derive from a single strain, and this limitation must be considered when generalizing these findings to all clinical isolates.

### 5.2 *In vitro* antimicrobial susceptibility

The first comprehensive antimicrobial susceptibility test (AST) data for *L. clevelandensis* were reported by Goldenberger et al. in 2019 (Goldenberger et al., 2019), determining minimum inhibitory concentrations (MICs) for a range of antimicrobial agents (**Table 3**). The strain grew well over 4 to 5 days under anaerobic conditions. In contrast to Müller-Hinton or Brucella agar, Columbia sheep’s-blood medium facilitated good and reliable growth. Therefore, the AST was evaluated on this medium. AST was executed twice independently using the gradient diffusion technology (MIC Test Strip; Liofilchem) against 18 antimicrobial agents. The plates were incubated anaerobically at 36 °C and were read after 3, 5 and 7 days using a binocular microscope and by eye (after 7 days only). All quality controls using Bacteroides fragilis ATCC 25285 were within the MIC ranges of listed antimicrobial agents in the Clinical and Laboratory Standards Institute guidelines. Susceptibility testing demonstrated low to very low MICs for all antimicrobial agents tested (**Table 3**).

**Table 3:** Antimicrobial susceptibility results from *L. clevelandensis* strain USB603019-18.

Antimicrobial agent	MIC value^#^ (mg/L)	Susceptibility interpretation
Penicillin	0.002	Susceptible
Amoxicillin/clavulanic acid	<0.016	Susceptible
Piperacillin/tazobactam	<0.016	Susceptible
Ceftriaxone	0.023	Susceptible
Cefuroxime	<0.016	Susceptible
Cefepime	0.016	Susceptible
Imipenem	<0.002	Susceptible
Erythromycin	0.032	Susceptible
Clindamycin	0.064	Susceptible
Tetracycline	0.047	Susceptible
Tigecycline	0.023	Susceptible
Ciprofloxacin	0.047	Susceptible
Moxifloxacin	0.094	Susceptible
Vancomycin	0.190	Susceptible
Gentamicin	0.380	Susceptible
Rifampicin	<0.002	Susceptible
Linezolid	0.094	Susceptible
Daptomycin	0.064	Susceptible

#value based on two parallel tests.

These data collectively demonstrate that *L. clevelandensis* strain USB603019–18 exhibited low to very low minimum inhibitory concentrations (MICs) for all antimicrobial agents tested, including penicillins, β-lactam/β-lactamase inhibitor combinations, cephalosporins (ceftriaxone, cefuroxime, cefepime), carbapenems (imipenem), macrolides (erythromycin), lincosamides (clindamycin), tetracyclines (tetracycline, tigecycline), fluoroquinolones (ciprofloxacin, moxifloxacin), vancomycin, rifampicin, linezolid, daptomycin, and gentamicin. Notably, the MIC for gentamicin was 0.38 mg/L, which is not indicative of intrinsic resistance. Whole-genome sequencing revealed no known acquired antimicrobial resistance or virulence genes, corroborating the phenotypic susceptibility findings (Goldenberger et al., 2019). These results, combined with clinical evidence in **Table 2**, suggest that *L. clevelandensis* is potentially susceptible to a broad range of antimicrobial agents *in vitro*. Clinically, amoxicillin/clavulanate (AMC) has been used successfully in multiple reported cases (**Table 2**). Therefore, aminoglycosides are not contraindicated based on available *in vitro* data, although their clinical efficacy against this strictly anaerobic organism remains unproven.

### 5.2 Clinical treatment strategy

The treatment of *L. clevelandensis* infection rests on two pillars: adequate source control through surgical or percutaneous drainage, and targeted antimicrobial therapy. Published clinical experience supports the following general approach:

#### 5.2.1 Source control

Surgical debridement, incision and drainage, or percutaneous drainage of abscesses is almost universally required in addition to antimicrobial therapy. This is particularly true for large abscesses, vascular graft infections (where graft removal may be necessary), and deep visceral infections. In the vascular graft infection case series, all patients underwent surgical intervention alongside antimicrobial therapy.

#### 5.2.2 Antimicrobial regimens

Given the broad differential that includes other anaerobes and potentially mycobacteria, empiric regimens should ideally cover *L. clevelandensis*. A reasonable empiric approach for a culture-negative deep abscess in which *L. clevelandensis* is suspected includes a beta-lactam/beta-lactamase inhibitor combination (e.g., ampicillin-sulbactam or piperacillin-tazobactam) or a carbapenem (imipenem or meropenem), optionally with the addition of metronidazole for enhanced anaerobic coverage.

Once *L. clevelandensis* is confirmed, therapy can be streamlined to one of the following oral or intravenous agents: amoxicillin-clavulanic acid (preferred oral agent), clindamycin (oral, useful for outpatient therapy), or ceftriaxone (intravenous, followed by oral step-down). Metronidazole is effective but its use as monotherapy is less well-documented in clinical cases.

#### 5.2.3 Treatment duration

The optimal duration of therapy has not been rigorously studied. Based on clinical experience, a duration of 4–8 weeks is commonly employed for soft tissue infections, while vascular graft infections and CNS infections typically require 3–6 months of antimicrobial therapy, often with prolonged suppressive regimens for prosthetic material infections that cannot be completely removed.

### 5.3 Clinical outcomes

The prognosis of patients with *L. clevelandensis* infection is generally favorable, and most achieve cure after abscess drainage and prolonged antibiotic therapy (Gonzales Zamora et al., 2020; Zhou et al., 2023; Liu et al., 2025; Hennen et al., 2026). Overall, the prognosis of patients with *L. clevelandensis* infection is favorable when the diagnosis is established promptly and appropriate treatment is initiated. Reported cure rates exceed 80% with combined surgical and antimicrobial therapy. Delayed diagnosis — primarily attributable to the organism’s fastidiousness and the failure of conventional culture — is the most significant predictor of poor outcomes, as it leads to prolonged ineffective empiric therapy and ongoing tissue damage. Immunocompromised patients and those with deep visceral or CNS involvement tend to require longer treatment courses.

## 6 Future perspectives

Several critical knowledge gaps remain and represent priorities for future investigation:

1. Pathogenesis and virulence: The mechanisms by which *L. clevelandensis* transitions from commensal to pathogen are essentially unknown. Comparative genomic studies and functional assays are needed to identify putative virulence factors, particularly those that may mediate tissue invasion and abscess formation.
2. Microbiome ecology: Further characterization of the cutaneous and gastrointestinal reservoirs of *L. clevelandensis*, including its prevalence and ecological role in conditions such as hidradenitis suppurativa, diabetic foot infections, and chronic wound infections, may provide important insights into its role as an opportunistic pathogen.
3. Diagnostic development: The inclusion of *L. clevelandensis* in commercial MALDI-TOF MS reference databases (e.g., Bruker Biotyper, VITEK MS) would represent a major advance in routine diagnostic capacity. Development of species-specific PCR assays for clinical use would also significantly improve time-to-diagnosis compared to current sequencing-based approaches.
4. Clinical breakpoints: Formal establishment of CLSI species-specific or general anaerobic breakpoints for *L. clevelandensis* is urgently needed to guide evidence-based therapeutic decisions and antimicrobial stewardship efforts.
5. Novel species and zoonotic potential: The recent discovery of ‘Ca.’ Lawsonella tjsk and the identification of *L. clevelandensis* in a canine infection warrant further investigation into the potential for novel *Lawsonella* species to cause human disease and possible zoonotic transmission pathways.
6. Tissue tropism and commensal-to-pathogen conversion: The striking predilection for fat-rich tissues (breast, abdomen, subcutis) observed in clinical cases demands mechanistic investigation. Future research should explore the lipid-metabolizing capabilities of *L. clevelandensis*, the role of its mycolic acid-containing cell wall in immune evasion, and the host factors that permit its transition from a harmless skin commensal to an invasive pathogen. Functional studies using *in vitro* infection models, transcriptomic profiling during host interaction, and, if feasible, the development of animal models will be essential to unravel these processes. Such knowledge could ultimately inform preventive strategies, particularly for surgical or cosmetic procedures involving adipose tissue.

## 7 Discussion

Accumulating evidence from the past decade establishes *L. clevelandensis* as a genuine opportunistic pathogen that exploits specific host vulnerabilities. Analysis of all documented cases (**Table 2**) reveals a consistent theme: the vast majority of patients have underlying conditions or iatrogenic exposures that compromise skin integrity or immune competence. The most frequently identified risk factors include diabetes mellitus (Harrington et al., 2013; Lindstrom et al., 2025), immunosuppressive therapy (rheumatoid arthritis, polymyalgia rheumatica, post-transplant), chronic inflammatory diseases (hidradenitis suppurativa, inflammatory bowel disease) (Chudy-Onwugaje et al., 2019; Lijia et al., 2024), and prior surgical procedures (spinal stabilization, vascular graft placement, mechanical heart valve, autologous fat grafting) (Harrington et al., 2013; Francuzik et al., 2018; Polak-Witka et al., 2021; Ramesh et al., 2021; Kumaria et al., 2023; Zhou et al., 2023). Even in apparently immunocompetent individuals, a history of recent trauma, cosmetic procedure, or indwelling prosthetic material is nearly always present (Chudy-Onwugaje et al., 2019; Zhou et al., 2023). These observations strongly support the “endogenous opportunist” model: *L. clevelandensis* resides as a commensal of normal skin and possibly the gastrointestinal tract (Rahlwes et al., 2019; Gonzales Zamora et al., 2020; Ahmed et al., 2021; Wang et al., 2023); when the epithelial barrier is breached and the local microenvironment becomes anaerobic and lipid-rich, the bacterium invades deeper tissues, leading to abscess formation.

Beyond the epidemiological association with host vulnerabilities, the striking tropism of *L. clevelandensis* for fat-rich tissues—most notably breast, abdomen, and subcutis—raises unresolved biological questions. In the 18 reviewed cases, adult females predominantly presented with breast abscesses (6/8, 75%) (**Table 2**), far exceeding expected frequency and suggesting an active preference for lipid-enriched microenvironments. This tissue selectivity echoes other Corynebacterineae members, such as *Mycobacterium tuberculosis*, which exploit host lipids as carbon sources (Rahlwes et al., 2019). Although sequenced genomes lack classical virulence genes, the presence of mycolic acids and meso-diaminopimelic acid in the cell wall implies potential lipid-processing capabilities that may confer a survival advantage in adipose niches. We hypothesize a multi-step transition from commensal to pathogen: epithelial barrier disruption, inoculation into anaerobic lipid-rich environments, immune evasion—possibly aided by the acid-fast cell wall resisting phagolysosomal degradation—and persistent abscess formation driven by host inflammation. However, the absence of functional genetic tools or animal models leaves these mechanisms speculative. Elucidating this commensal-to-pathogen conversion is clinically relevant: it may inform risk stratification, guide preventive strategies in surgical/cosmetic procedures involving adipose tissue, and potentially uncover novel therapeutic targets that disrupt lipid-dependent survival pathways.

The organism’s distinct microbiological traits—strict anaerobiosis, partial acid-fast staining, extremely slow growth, and absence from commercial MALDI-TOF databases—pose substantial diagnostic challenges. Clinicians confronted with an acid-fast bacillus in a deep abscess may erroneously initiate anti-tuberculous therapy, while routine anaerobic cultures often turn negative due to insufficient incubation time. The 2026 brain abscess case (Hennen et al., 2026) and the 2024 widespread soft tissue infection case (Lijia et al., 2024) both illustrate that molecular methods, particularly mNGS and 16S rRNA sequencing, are now indispensable for timely diagnosis. Indeed, mNGS has transformed *L. clevelandensis* from a rarely recognized organism into a detectable pathogen, and its wider availability will likely uncover many more cases across diverse anatomical sites.

Treatment outcomes are generally favorable when source control (surgical or percutaneous drainage) is combined with appropriate antibiotics. Based on available susceptibility data (Goldenberger et al., 2019), β-lactam/β-lactamase inhibitors (amoxicillin-clavulanate, piperacillin-tazobactam), carbapenems, metronidazole, and clindamycin are reliable choices. Prolonged therapy (4–8 weeks for soft tissue; 3–6 months for vascular graft or CNS infections) is recommended due to the very low growth rate of *L. clevelandensis*, especially in immunocompromised hosts or those with retained prosthetic material. Delayed diagnosis remains the strongest predictor of poor outcome.

In conclusion, *L. clevelandensis* epitomizes an emerging paradigm: a normal flora component that “bites deep” only when predisposing conditions align. Future research should focus on incorporating this organism into routine MALDI-TOF databases, establishing CLSI/EUCAST breakpoints, and elucidating its virulence mechanisms—particularly its affinity for adipose-rich tissues. Only then can we transform current reactive diagnosis into proactive clinical awareness.
